# Using genetics to understand the causal influence of higher BMI on depression

**DOI:** 10.1093/ije/dyy223

**Published:** 2018-11-13

**Authors:** Jessica Tyrrell, Anwar Mulugeta, Andrew R Wood, Ang Zhou, Robin N Beaumont, Marcus A Tuke, Samuel E Jones, Katherine S Ruth, Hanieh Yaghootkar, Seth Sharp, William D Thompson, Yingjie Ji, Jamie Harrison, Rachel M Freathy, Anna Murray, Michael N Weedon, Cathryn Lewis, Timothy M Frayling, Elina Hyppönen

**Affiliations:** 1 Genetics of Complex Traits, University of Exeter Medical School, Royal Devon & Exeter Hospital, Exeter, UK; 2 Australian Centre for Precision Health, University of South Australia Cancer Research Institute, Adelaide, SA, Australia; 3 Social, Genetic and Developmental Psychiatry Centre, Institute of Psychiatry, Psychology and Neuroscience, King’s College London, London, UK

**Keywords:** Body mass index, depression, Mendelian randomization, UK Biobank

## Abstract

**Background:**

Depression is more common in obese than non-obese individuals, especially in women, but the causal relationship between obesity and depression is complex and uncertain. Previous studies have used genetic variants associated with BMI to provide evidence that higher body mass index (BMI) causes depression, but have not tested whether this relationship is driven by the metabolic consequences of BMI nor for differences between men and women.

**Methods:**

We performed a Mendelian randomization study using 48 791 individuals with depression and 291 995 controls in the UK Biobank, to test for causal effects of higher BMI on depression (defined using self-report and Hospital Episode data). We used two genetic instruments, both representing higher BMI, but one with and one without its adverse metabolic consequences, in an attempt to ‘uncouple’ the psychological component of obesity from the metabolic consequences. We further tested causal relationships in men and women separately, and using subsets of BMI variants from known physiological pathways.

**Results:**

Higher BMI was strongly associated with higher odds of depression, especially in women. Mendelian randomization provided evidence that higher BMI partly causes depression. Using a 73-variant BMI genetic risk score, a genetically determined one standard deviation (1 SD) higher BMI (4.9 kg/m^2^) was associated with higher odds of depression in all individuals [odds ratio (OR): 1.18, 95% confidence interval (CI): 1.09, 1.28, *P* = 0.00007) and women only (OR: 1.24, 95% CI: 1.11, 1.39, *P* = 0.0001). Meta-analysis with 45 591 depression cases and 97 647 controls from the Psychiatric Genomics Consortium (PGC) strengthened the statistical confidence of the findings in all individuals. Similar effect size estimates were obtained using different Mendelian randomization methods, although not all reached *P *< 0.05. Using a metabolically favourable adiposity genetic risk score, and meta-analysing data from the UK biobank and PGC, a genetically determined 1 SD higher BMI (4.9 kg/m^2^) was associated with higher odds of depression in all individuals (OR: 1.26, 95% CI: 1.06, 1.50], *P *=* *0.010), but with weaker statistical confidence.

**Conclusions:**

Higher BMI, with and without its adverse metabolic consequences, is likely to have a causal role in determining the likelihood of an individual developing depression.

**Key Messages**

- This study provides evidence for the causal role of high BMI in depression, using individual-level data from the UK Biobank and summary statistics from the Psychiatric Genomics Consortium. It represents the largest set of data used to address this question.
- Using genetic variants associated with high BMI but a favourable metabolic profile, we demonstrate evidence for a causal relationship between high BMI and depression in the absence of adverse metabolic effects.
- The availability of individual-level statistics from the UK Biobank enabled us to compare the relationship in males and females separately.
- Negative control Mendelian randomization tests were also performed to explore the potential for residual confounding in the data.

## Introduction

Obesity and depression are two global health problems, that are estimated to cost the global economy trillions of dollars per annum.^1^^,^^2^ Higher body mass index (BMI) is observationally associated with higher odds of depression.^3^^,^^4^ These associations tend to be stronger in women than men, with a U-shaped relationship often observed in men.^5^^,^^6^ It is important to understand whether obesity causes depression, to optimize public health and medical intervention planning. Weight loss is difficult to achieve and maintain, and even more so for people with depression.^7^^,^^8^ There is much debate within the literature about the directionality of the relationship between obesity and depression, with conflicting evidence from different studies including meta-analyses and prospective data. These studies include those suggesting that: (i) obesity causes depression;^9^ (ii) depression causes obesity;^10^ or (iii) there is a reciprocal link between the two.^11^ However, determining causality is not trivial, especially when most observational associations will be confounded or biased.

**Figure 1.**
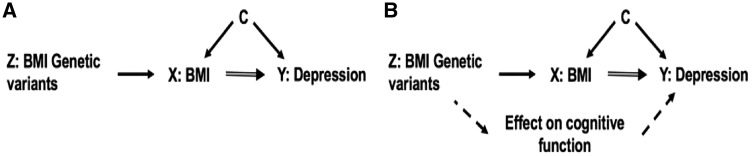
The principles of Mendelian randomization and key MR assumptions which are: (i) the genetic instrument (Z) is robustly related to the risk factor of interest (X); (ii) Z is not associated with confounders (C) of the X-outcome (Y) association; and (iii) there is no path from Z to Y other than through X (part a). Part b shows how this may be violated.

A genetic approach, Mendelian randomization (MR) (Figure 1), can be used to test for a causal relationship between higher BMI and depression. Genetic variants associated with BMI can act as unconfounded proxies because inherited genetic variation is randomly allocated at conception. A number of studies have used this method to investigate if BMI causally influences depression. These studies were limited by either relatively small sample sizes for MR, or by a reliance on summary statistics from large genome-wide association study (GWAS) consortia. The reliance on summary statistics from GWAS means that stratified analyses, for example by sex, are not possible. Previous studies include those using the *FTO* variant^12^ and a 32-single nucleotide polymorphism (SNP) BMI genetic risk score (GRS).^13^ Two more recent studies used statistics from up to 90 variants associated with BMI and the most recent GWAS studies of depression. These studies included 9240 cases and 9519 controls^14^ and 135 458 cases and 344 901 controls, respectively,^15^ and provided further evidence for the causal role of BMI in depression. However, these previous studies were not able to answer more detailed questions about the potential causal relationship between BMI and depression. First, no studies have used genetics to test the causal role of BMI in men and women separately, an important consideration given the different observational associations and well-known social and cultural differences of body image between men and women.^16^^,^^17^ These differences include those at the ends of the BMI spectrum, with very thin men more likely to be depressed than normal weight men and very thin women (creating a U-shaped relationship in men^5^ possibly due to illness). Second, MR studies to date have not explored the hypothesis that obesity, or the perception of body size, in childhood could influence depression in later life. Finally, previous studies have not attempted to uncouple the potential psychological aspects of the BMI-depression relationship from physiological aspects—something that is very difficult to do without a genetic approach. Previous studies have suggested that physiological as well as psychological factors could cause depression—for example poorer overall health and adverse metabolic factors such as inflammation.^18^^,^^19^

Here, we used Mendelian randomization to test the hypothesis that causal pathways link BMI to higher odds of depression and explore the role of both metabolic and psychological components of obesity. We used data from the UK Biobank, starting from 451 099 individuals of European ancestry, of whom we classified 340 786 as either a depression case or control. We used two genetic instruments, both representing higher BMI, but one with and one without its adverse metabolic consequences, in an attempt to ‘uncouple’ the psychological component of obesity from the metabolic consequences (metabolically ‘unfavourable’ and ‘favourable’ adiposity^20^). We tested effects in men and women separately, used a subset of BMI-associated variants to test effects more likely to be specific to neuronal pathways and examined the relationship between perceived childhood size and depression.

## Methods

### UK Biobank

The UK Biobank is a study of 500 000 individuals aged between 37 and 73 years (with 99.5% between 40 and 69 years), recruited from across the UK in 2006–10. The study is described in detail elsewhere.^21^ Briefly, participants provided a range of information via questionnaires and interviews (e.g. demographics, health status), anthropometric measures and blood pressure readings; blood, urine and saliva samples were taken for future analysis, and participants agreed to have their health followed over time via hospital record linkage and follow-ups. Genetics were available for all individuals. SNP genotypes were generated from the Affymetrix Axiom UK Biobank array (∼450 000 individuals) and the UKBiLEVE array (∼50 000 individuals). This dataset underwent extensive central quality control [http://biobank.ctsu.ox.ac.uk]. We based our study on 451 099 individuals of White European descent, as defined by principal components analysis (PCA). Briefly, principal components were generated in the 1000 Genomes Cohort using high-confidence SNPs to obtain their individual loadings. These loadings were then used to project all of the UK Biobank samples into the same principal component space, and individuals were then clustered using principal components 1 to 4. Participants were removed if they had subsequently withdrawn from the study (*n* = 7) or if they were sex mismatches (*n* = 348; self-reported sex did not match genetic sex). We also used a subset of unrelated individuals (*n* = 379 768). The unrelated individuals were defined from the 451 099 individuals of White European descent, and the KING Kinship matrix was used to separate out related individuals (up to third degree). An optimal list of unrelated individuals was generated to allow maximum numbers of individuals to be included. Ancestral principal components were then generated within these identified individuals for use in subsequent analyses.

### Patient involvement

This study was conducted using the UK Biobank resource, which has details on patient and public involvement available online at [http://www.ukbiobank.ac.uk/about-biobank-uk/] and [https://www.ukbiobank.ac.uk/wp-content/uploads/2011/07/Summary-EGF-consultation.pdf?phpMyAdmin=trmKQlYdjjnQIgJ%2CfAzikMhEnx6]. No patients were specifically involved in setting the research question or the outcome measures, nor were they involved in developing plans for recruitment, design or implementation of this study. No patients were asked to advise on interpretation or writing up of results. There are no specific plans to disseminate the results of the research to study participants, but the UK Biobank disseminates key findings from projects on its website.

### Exposure and outcome measures

In this study, we investigated the causal role of BMI on depression; BMI is therefore the exposure and depression is the outcome. We used genetic instruments to investigate the role of higher BMI on depression, with and without its adverse effects, including inflammation, insulin resistance and metabolic diseases such as type 2 diabetes, hypertension and coronary artery disease.

#### Body mass index

BMI was calculated for all participants from measured weight (kg)/height (m)^2^. BMI was available for 340 786 individuals with genetic data available, whom we classified as depression cases or controls. BMI was inverse-normalized before analysis. The BMI variable was validated and demonstrated to associate with known demographics including age, sex, socioeconomic position and type 2 diabetes status (Supplementary Table 1, available as Supplementary data at *IJE* online). We also created a binary obesity variable where we classified individuals as normal BMI (measured BMI between 18.5 and 24.9 kg/m^2^) and obese individuals (BMI greater than 30 kg/m^2^).

#### Depression

The main depression measure used in these analyses combined data from the self-report questionnaire in UK Biobank and the Hospital Episode Statistic data. Individuals were considered a case if they met one or more of the following criteria:

- self-reported seeing a GP for nerves/anxiety or depression AND reported at least a 2-week duration of depression or unenthusiasm;
- self-reported seeing a psychiatrist for nerves/anxiety or depression AND reported at least a 2-week duration of depression or unenthusiasm;
- had the following ICD-10 codes in the Hospital Episode Statistics: F33 representing recurrent major depressive disorder (MDD) or F32 representing single-episode MDD.

**Figure 2.**
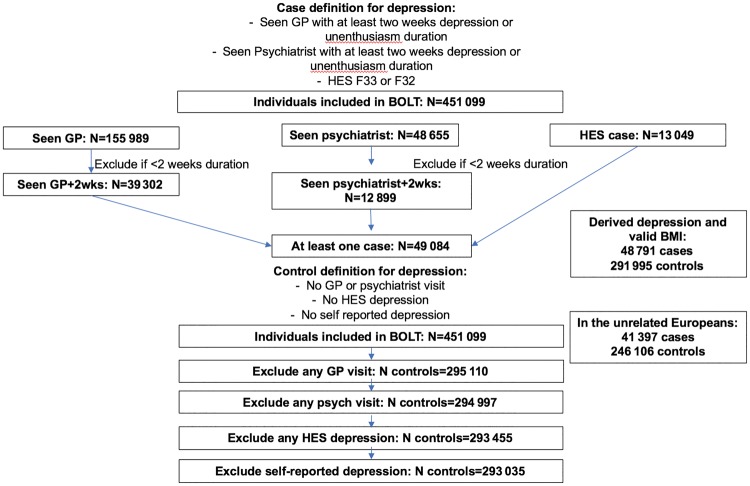
Flow chart explaining the derivation of depression cases and controls in the UK Biobank.

By combining these criteria, we had 48 791 cases (41 397 in the analyses of unrelated participants) with a valid BMI measure (Figure 2).

Controls were defined as individuals not reporting ever visiting a GP or psychiatrist for nerves/anxiety or self-reporting depression in the non-cancer illness variable in the UK Biobank and not having an ICD-10 code relating to MDD. This resulted in 291 995 controls (246 106 in unrelated) with a valid BMI measure. Basic characteristics of the cases and controls are described in Table 1 and in Supplementary Table 2, available as Supplementary data at *IJE* online.

**Table 1.** Demographics and lifestyle characteristics in depression cases and controls in all individuals, men only and women only

Demographic	All individuals			Men only			Women only		
	Depression cases	Controls	*P* ^a^	Depression cases	Controls	*P* ^a^	Depression cases	Controls	*P* ^a^
*N*	41 397	246 106		15175	127 709		26 222	118 397	
Mean age at recruitment in years (SD)	56.5 (7.9)	57.5 (8.1)	<1 x 10^−15^	57.1 (7.9)	57.6 (8.2)	<1 x 10^−15^	56.2 (7.8)	57.3 (8.0)	<1 x 10^−15^
Male, *n* (%)	15 175 (36.7)	127 709 (51.9)	<1 x 10^−15^	NA			NA		
Mean Townsend deprivation index (SD)	−0.98 (3.1)	−1.64 (2.9)	<1 x 10^−15^	−0.82 (3.2)	−1.60 (2.9)	<1 x 10^−15^	−1.07 (3.0)	−1.69 (2.8)	<1 x 10^−15^
Mean physical activity level (SD)^b^	7.35 (1.2)	7.44 (1.1)	<1 x 10^−15^	7.37 (1.2)	7.47 (1.1)	<1 x 10^−15^	7.34 (1.2)	7.40 (1.1)	2x10^−14^
Smoking status			<1 x 10^−15^			<1 x 10^−15^			<1 x 10^−15^
Never, *n* (%)	20 052 (48.4)	138 104 (56.1)		6412 (42.3)	64 353 (50.4)		13 604 (52.0)	73 751 (62.3)	
Former, *n* (%)	15 358 (37.1)	84 543 (34.4)		6158 (40.6)	48 806 (38.2)		9200 (335.1)	35 737 (30.2)	
Current, *n* (%)	5356 (12.9)	20 379 (8.3)		2337 (15.4)	12 617 (9.9)		3019 (11.5)	7762 (6.5)	
Missing, *n* (%)	631 (1.5)	3080 (1.3)		268 (1.8)	1933 (1.5)		363 (1.4)	1147 (1.0)	
Mean body mass index in kg/m^2^ (SD)	27.9 (5.4)	27.2 (4.6)	<1 x 10^−15^	28.2 (4.7)	27.8 (4.1)	<1 x 10^−15^	27.8 (5.7)	26.7 (4.9)	<1 x 10^−15^
Reported body size at age 10			0.34			0.006			0.40
Smaller than average, *n* (%)	14 022 (33.9)	77 794 (31.6)		5520 (36.4)	42 486 (33.3)		8502 (32.4)	35 308 (29.8)	
Average, *n* (%)	19 085 (46.1)	127 445 (51.8)		7029 (46.3)	65 831 (51.5)		12 056 (46.0)	61 614 (52.0)	
Larger than average, *n* (%)	7690 (18.6)	36 582 (14.9)		2343 (15.4)	16 808 (13.2)		5347 (20.4)	19 774 (16.7)	
Missing, *n* (%)	600 (1.4)	4285 (1.7)		283 (1.9)	2584 (2.0)		317 (1.2)	1701 (1.5)	
Parents depressed, *n* (%)	6647 (16.1)	15 833 (6.4)	<1 x 10^−15^	2245 (14.8)	7295 (5.7)	<1 x 10^−15^	4402 (16.8)	8538 (7.2)	<1 x 10^−15^

a Comparison of cases and controls using linear (for continuous) and logistic (for binary) regression models.

b From self-reported data and based on the International Physical Activity Questionnaire.

In this case-control analysis, we used participants who did not have any indication for history of depression as the control group, and excluded participants with unclear status and/or likely mild depression. Of these 110 000 individuals who were neither included in depression case nor in control group, 35% were men, 12% were smokers and 8% identified themselves as ‘non-drinkers’. Around 13% of these individuals reported that they had had a doctor-diagnosed depression. Nearly all (99.7%) had seen a GP or psychiatrist for depression, anxiety, nervousness or tension, but either had not experienced prolonged periods of depression and/or unenthusiasm (8%) or had this information missing (92%).

A number of more stringent measures were considered in sensitivity analyses, with the depression measure further characterized as: (i) single episode; or (ii) recurrent, based on the classification in the ICD-10 code data and/or using the self-reported number of occurrences variable in the UK Biobank. Finally, a depression measure that only used the HES data was also considered.

### Observational associations

We regressed depression status against BMI using logistic regression models. We adjusted these for age, sex and assessment centre. Models were then further adjusted for a measure of socioeconomic position (using the Townsend deprivation index), smoking, alcohol consumption and physical activity measures (using data from the international physical activity questionnaire). Similar analyses were conducted for self-perceived weight status at age 10 years, with further adjustment for adult BMI.

### Genetic variants

We selected genetic variants from UK Biobank’s imputation dataset. Variants were excluded if the genotype probability was <0.9. Details of the imputation quality are given in Supplementary Table 3, available as Supplementary data at *IJE* online.

#### Genetic variants associated with BMI

We selected 73 of 76 common genetic variants that were associated with BMI at genome-wide significance in the GIANT consortium in studies of up to 339 224 people (Supplementary Table 3, available as Supplementary data at *IJE* online).^22^ We limited the BMI variants to those that were associated with BMI in the analysis of all people of European ancestry, and did not include those that reached genome-wide levels of statistical confidence in only one sex or one stratum. We also excluded variants if they were known to be classified as a secondary signal within a locus. In the primary analysis, three variants were excluded from the score because they were known to have pleiotropic effects on other traits [rs11030104 (*BDNF* reward phenotypes including smoking), rs13107325 (*SLC39A8* lipids, blood pressure), rs3888190 (*SH2B1* multiple traits)]. These three variants were defined as pleiotropic because they had larger effects on a third trait than their effects on BMI, meaning they are very likely acting on non-BMI related pathways. We repeated the MR tests with these three variants back in as a sensitivity analysis.

#### Neuronal and non-neuronal BMI genetic instruments

The 73 BMI SNPs were further separated into two subgroups—a neuronal and a non-neuronal group, based on their presence in or near genes enriched in specific pathways as summarized in Supplementary Table 3 of the Locke *et al*., 2015 paper^22^ (Supplementary Table 3, available as Supplementary data at *IJE* online). The neuronal group included 43 SNPs implicated in either neuronal development, neuronal expression, neurotransmission and/or hypothalamic expression pathways. The remaining 30 BMI SNPs had not been implicated in the above pathways and were categorized into the non-neuronal group.

#### Favourable adiposity genetic variants

We selected 14 common variants (Supplementary Table 3, available as Supplementary data at *IJE* online) that were associated with higher body fat percentage, but lower risk of metabolic disease.^20^^,^^23^ The 14 variants explained 0.2% of the variance in BMI in the UK Biobank participants. These variants were defined as follows. We performed a genome-wide association study of body fat percentage, as measured by impedance, in the UK Biobank. In parallel we performed a multivariate genome-wide association study of seven traits using summary statistics from published GWAS—body fat percentage,^24^ high-density lipoprotein (HDL) cholesterol,^25^ triglycerides,^25^ sex hormone binding globulin,^26^ adiponectin,^27^ fasting insulin^28^ and alanine aminotransferase (ALT).^29^ Cross-tabulation of the variants associated with body fat percentage and the variants associated with the multivariate outcome revealed 33 variants associated with multiple metabolic outcomes. A cluster analysis of these 33 variants revealed 14 that clustered together because alleles associated with higher body fat percentage were associated with a favourable metabolic profile of the other six markers (e.g. the allele associated with higher body fat percentage was associated with higher HDL cholesterol and lower triglycerides).

Individual variants were recoded as 0, 1 and 2, according to the number of adult BMI or favourable adiposity increasing alleles. Weighted GRS were created using the adult BMI and favourable adiposity. Each variant was weighted by its effect size (β-coefficient) obtained from the primary GWAS that did not include any data from the UK Biobank.^20^^,^^22^ The weighted score was rescaled to reflect the number of trait-increasing alleles (Equation 2).

(Equation 1) $$Weighted\;score=\;\beta_{1}\;x\;{SNP}_{1}+\;\beta_{2}\;x\;{SNP}_{2\;}+\cdots \beta_{n}\;x\;{SNP}_{n}$$

(Equation 2) $$Weighted\;genetic\;risk\;score=\;\frac{weighted\;score\;x\;number\;of\;SNPs}{sum\;of\;the\;\beta \;coefficients}$$

### Mendelian randomization

We used Mendelian randomization to investigate causal pathways between BMI and depression. Mendelian randomization relies on several assumptions as outlined below:^30^

- the BMI and favourable adiposity genetic risk scores are robustly associated with measured BMI;
- the BMI and favourable adiposity genetic risk scores are not associated, independently of their effects on BMI, with confounding factors that bias conventional epidemiological associations between depression and obesity.

In this study, we employed several Mendelian sandomization approaches, first the standard one-sample instrumental variable analyses using the genetic risk scores in the unrelated data set of 287 503 individuals. Second, we investigated the causal relationship using a two-sample approach in both the unrelated (*n* = 287 503) and related (*n* = 340 786) individuals. In this step, we tested if our results were robust to any potential influence of population stratification by using linear mixed models approach as implemented in the software BOLT-LMM (version 2.3^31^). This approach corrects for all levels of interindividual correlation of genotypes due to relatedness, from close to distant relatives.

#### One-sample Mendelian randomization: instrumental variable analysis

We employed the two-stage least-squares regression estimator method that uses predicted levels of BMI per genotype and regresses the depression outcome against these predicted values. The instrumental variable analysis was performed in two stages, as all our outcomes were binary. First, we assessed the association between the BMI or favourable adiposity GRS and BMI. The predicted values and residuals from this regression model were saved. Second, the predicted values from stage 1 were used as the independent variable (reflecting an unconfounded estimate of variation in BMI) and depression as the dependent variable in a logistic regression model. Robust standard errors were used to correct for the uncertainty in the estimate.

#### Two-sample Mendelian randomization approaches

We also employed a two-sample MR approach to analyse results in the larger dataset corrected for relatedness using BOLT-LMM. The depression outcome was run through BOLT-LMM as a genome-wide association scan. The BMI and favourable adiposity genetic variants were then extracted. We performed inverse variance weighted (IVW) instrumental variable analysis and two methods that are more robust to the potential violations of the standard instrumental variable assumptions (MR-Egger^32^ and Median MR^33^) The two-sample approach regresses the effect sizes of variant-outcome associations (here BMI or favourable adiposity variants versus depression) against effect sizes of the variant-risk factor associations (here BMI or favourable adiposity variants versus BMI). The variant-risk factor associations were taken from the primary GWAS of BMI^22^ and from the primary GWAS of body fat percentage for the 14 favourable adiposity variants.^20^^,^^24^ We also extracted association statistics for the 73 BMI and 14 favourable adiposity variants from the recent GWAS of depression by the Psychiatric Genomics Consortium (PGC; Supplementary material, available as Supplementary data at *IJE* online) to replicate our two-sample MR analyses in an independent sample of 45 591 depression cases and 97 647 controls, which excluded the UK Biobank samples and 23&Me samples from the primary PGC analyses.^15^

The IVW approach assumes that there is no horizontal pleiotropy (under a fixed effect model) or, if implemented under a random effects model after detecting heterogeneity among the causal estimates, that:

- the strength of the association of the genetic instruments with the risk factor is not correlated with the magnitude of the pleiotropic effects; and
- the pleiotropic effects have an average value of zero.

The MR-Egger uses a weighted regression with an unconstrained intercept to remove the assumption that all genetic variants are valid instrumental variables, and is therefore less susceptible to confounding from potentially pleiotropic variants that have a stronger effect on the outcome compared with their effect on the primary traits. The Median-MR method takes the median instrumental variable from all variants included, and is robust when up to (but not including) 50% of the genetic variants are invalid. Given these different assumptions, if all methods are broadly consistent this strengthens our causal inference. Details of the R code for the two-sample IVW, MR-Egger and Median-MR analyses are provided in Bowden *et al.*, 2015 and 2016.^33^^,^^34^

Assuming that both neuronal and non-neuronal genetic risk scores instrumented the same exposure, BMI, we used an over-identification test^35^ to check whether the causal estimates from these two genetic risk scores were consistent (i.e. both of the GRS are valid genetic instruments). Rejecting this hypothesis may indicate the effect of genetic risk score on depression through a mechanism other than through BMI. For this particular analysis, we used ‘*ivprobit*’ command- based two-stage regression followed by an over-identification test (using ‘*overid’* Stata command).

### Differences between men and women

To test the hypothesis that the effects of BMI on depression may differ in males and females, we repeated observational and genetic analyses separately in each sex. The selected BMI and favourable adiposity genetic variants have very similar effects in men and women, and therefore the same genetic variants and risk scores were used in all participants, in males only and in females only. The beta values for males and females were compared using Fisher’s z-score method (Equation 3).^36^

$$z=\frac{\beta_{1}-\beta_{2}}{\sqrt{{{SE}_{1}}^{2}+{{SE}_{2}}^{2}}}$$

### Sensitivity analysis

We performed six sensitivity analyses. We repeated our analysis, first using more stringent measures of depression by: (i) further categorizing the depression into single-episode depression and recurrent depression, using the number of depression episodes in UK Biobank; (ii) restricting individuals to those with a Hospital Episode Statistic (HES) record, of whom 10 939 had a primary or secondary ICD-10 code of single-episode or recurrent MDD (F32 and F33, respectively; 95% reported as a secondary diagnosis); and (iii) using the mental health questionnaire data available in 124 282, where participants were asked ‘Have you been diagnosed with depression by a professional, even if you don’t have it currently?’. Second, we repeated our analysis restricting to individuals self-reporting no other health problems [including cancers and non-cancer (e.g. type 2 diabetes and coronary artery disease) illnesses at baseline]. Third, we repeated our analysis excluding individuals reporting a family history of depression, as this was strongly associated with depression in our participants (OR: 2.74, 95% CI: 2.64, 2.82). Fourth, we repeated the analysis by excluding underweight (BMI <18.5 kg/m^2^) individuals. Finally, we repeated the primary MR test in all individuals with a 76 BMI SNP instrument that included the three variants we defined as pleiotropic.

#### Further tests to minimize the potential influence of pleiotropy

To objectively identify and exclude variants with potential pleiotropic effects, we tested the association of each BMI variant and each favourable adiposity variant against the depression measure. Variants with larger effects on the outcome than the exposure are unlikely to be specific instruments for the exposure. The odds ratio estimates were converted to standard deviation effect sizes and compared with the primary trait effect size for the variant (Supplementary Table 4, available as Supplementary data at *IJE* online).^37^ No SNPs were excluded based on these analyses.

#### Childhood BMI and depression later in life

Using a self-reported perceived body weight at age 10, we also investigated the influence of childhood BMI on depression later in life. The self-reported perceived body weight at age 10 was derived from the following question: ‘When you were 10 years old, compared to average would you describe yourself as:’, with the options: ‘Thinner’, ‘Plumper’, ‘About average’, ‘Prefer not to answer’ or ‘Don’t know’. The observational association between self-perceived weight status at age 10 and depression was adjusted for adult BMI.

#### Negative control tests

We also tested the validity of the Mendelian randomization approaches by performing a number of negative control experiments. These tests included looking at the observational and genetic BMI associations with: i) sun protection use; (ii) nitrogen dioxide pollution levels; and (iii) urban or rural home dwelling. These measures are strongly observationally associated with BMI, but BMI causing changes in sun protection use, nitrogen dioxide pollution levels or home location is less plausible.

## Results

The demographics of the 340 786 UK Biobank individuals with valid genetic data, BMI and whom we had classified as a depression case or control, are summarized in Table 1 and Supplementary Table 2, available as Supplementary data at *IJE* online.

### Confirmation of association between BMI genetic instruments and BMI in the UK Biobank

The BMI and favourable adiposity GRS were robustly associated with BMI, explaining 1.7% and 0.2% of the variance, respectively. The BMI GRS was also robustly associated with obesity, explaining 1.5% of the variance.

The BMI GRS was associated with several potential confounding factors. These included cigarette smoking and measures of socioeconomic position (including income and Townsend deprivation index). The associations with measures of socioeconomic position disappeared when adjusting for BMI (Supplementary Table 4, available as Supplementary data at *IJE* online), suggesting that BMI causes changes to socioeconomic position, consistent with previous Mendelian randomization analyses.^38^ The association between smoking and the BMI GRS weakened but remained statistically robust. The favourable adiposity GRS was not associated with any of the potential confounding factors tested (Supplementary Table 5, available as Supplementary data at *IJE* online).

### Higher BMI is associated with depression in the UK Biobank

**Figure 3.**
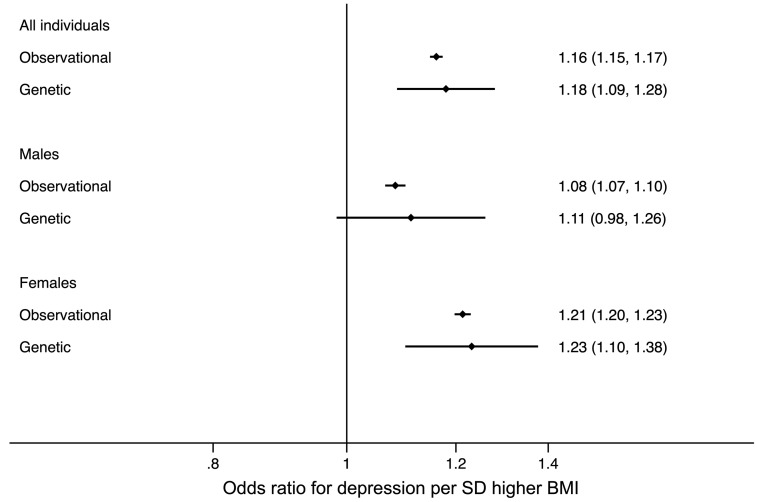
Forest plot of the observational and genetic associations between a 1-SD higher BMI and the odds of depression. The plots display the observational association (Observational) and the genetic association using the two-step instrumental variable analysis with the BMI genetic risk score (Genetic 1-sample).

Higher BMI was observationally associated with higher odds of depression (Table 2). A 1-SD (4.7 kg/m^2^) higher BMI was associated with a 1.16 (95% CI: 1.15, 1.17) higher odds of depression. This observational association was stronger in women than men (women: 1.21, 95% CI: 1.20, 1.23; men: 1.08, 95% CI: 1.07, 1.10, *P*_difference_ <1 x 10^-15^; Figure 3).

**Table 2.** Associations between higher BMI and depression, using logistic regression and instrumental variable analysis in the 287 503 unrelated UK Biobank individuals

			Observational^a^		Genetic^b^		Genetic: Egger^c^	
Instrument tested	Included individuals	*N* cases (controls)	Odds ratio (95% CI) of depression per SD higher BMI	*P*	Odds ratio (95% CI) of depression per SD higher BMI	*P*	Odds ratio (95% CI) of depression per SD higher BMI	*P*
BMI	All individuals	41 397 (246 106)	1.16 (1.15, 1.17)	<1x10^−15^	1.18 (1.09, 1.28)	7x10^−5^	1.24 (1.02, 1.50)	0.03
			*1.13 (1.11, 1.14)*	*<1x10^−15^*				
	Men only	15 175 (127 709)	1.08 (1.07, 1.10)	<1x10^−15^	1.11 (0.98, 1.26)	0.09	1.23 (0.94, 1.61)	0.13
			*1.07 (1.05, 1.09)*	*8x10^−11^*				
	Women only	26 222 (118 397)	1.21 (1.20, 1.23)	<1x10^−15^	1.23 (1.10, 1.38)	2x10^−4^	1.26 (1.01, 1.56)	0.04
			*1.18 (1.16, 1.20)*	*<1x10^−15^*				
Favourable adiposity	All individuals	41 397 (246 106)	1.16 (1.15, 1.17)	<1x10^−15^	1.52 (0.88, 2.61)	0.13	1.10 (0.60, 2.01)	0.77
			*1.13 (1.11, 1.14)*	*<1x10^−15^*				
	Men only	15 175 (127 709)	1.08 (1.07, 1.10)	<1x10^−15^	1.15 (0.47, 2.81)	0.75	0.52 (0.22, 1.23)	0.16
			*1.07 (1.05, 1.09)*	*8x10^−11^*				
	Women only	26 222 (118 397)	1.21 (1.20, 1.23)	<1x10^−15^	1.79 (0.90, 3.54)	0.09	1.71 (0.78, 3.78)	0.56
			*1.18 (1.16, 1.20)*	*<1x10^−15^*				

BMI analyses were repeated with additional variants^39^ and, although the inclusion of these variants narrowed the confidence intervals, it did not change the conclusions. Odds of depression (95% CI) in all, men only and women only was 1.12 (95% CI: 1.05, 1.19), 1.11 (95% CI: 1.01, 1.22) and 1.13 (95% CI: 1.04, 1.23), respectively.

a Age- and sex-adjusted associations and the further adjusted models in italics which included a measure of socioeconomic position (the Townsend deprivation index), smoking, alcohol consumption and physical activity measures (using data from the International Physical Activity Questionnaire) as covariates.

b Uses instrumental variable analysis and a two-step procedure for the binary outcomes using the BMI or favourable adiposity GRS. The F-statistic is >4705 in all individuals, >2466 in men only and >2313 in women only for BMI, and >86.5 in all individuals, >40.6 in men only and >44.6 in women only.

c Alternative genetic approach.^33^ Note full results for related and unrelated individuals are provided in Supplementary Table 5, available as Supplementary data at *IJE* online, for BMI, and Supplementary Table 7, available as Supplementary data at *IJE* online, for favourable adiposity.

Obese individuals were at 1.45 (95% CI: 1.41, 1.49) higher odds of depression than those of normal BMI, with stronger associations in women (OR: 1.59, 95% CI: 1.53, 1.64) than men (OR: 1.24, 95% CI: 1.19, 1.30).

### Mendelian randomization evidence that higher BMI causes depression

#### 

##### One-sample analysis in the UK Biobank

One-sample Mendelian randomization tests, using unrelated individuals of European ancestry from the UK Biobank, provided evidence for a causal role of higher BMI in depression. A genetically determined 1-SD (4.7 kg/m^2^) higher BMI was associated with higher odds of depression (OR: 1.18, 95% CI: 1.09, 1.28; Table 2 and Figure 3). The evidence for a gender difference in the causal association between BMI and depression did not reach *P* <0.05 (Figure 2), although the point estimates were higher in women than in men (1-SD higher BMI resulting in 1.23 higher odds of depression, 95% CI: 1.10, 1.38, in women and 1.11, 95% CI: 0.98, 1.26, in men, *P*_difference_ = 0.18).

Similar evidence was observed when genetically instrumented obesity was considered. Obese individuals were at higher odds of depression 1.11, 95% CI: 1.06, 1.16]. This was consistent in men (OR: 1.07, 95% CI: 1.00, 1.14]) and women (OR: 1.14, 95% CI: 1.06, 1.21]).

###### Two-sample analysis

**Figure 4.**
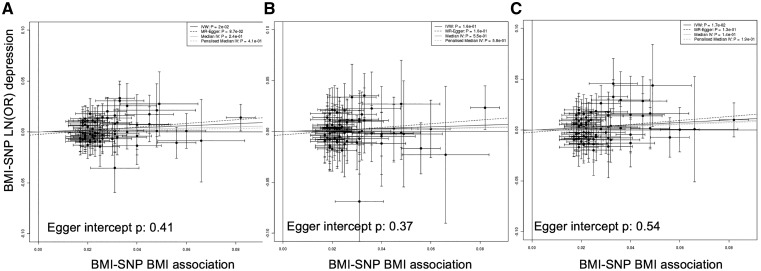
Plot of the individual BMI variant—BMI associations from the primary GWAS that did not include UK Biobank^22^ against the BMI variant—depression associations on natural log scale (LN(OH)) from related Europeans in the UK Biobank, in: A) all individuals; B) males only; and C) females only. The beta regression coefficients for inverse variance weighted (IVW) instrumental analysis (black solid), Egger-MR (black dash),^33^ median-IV (grey solid) and the penalized weight median IV (grey dash) are plotted. The Egger intercept *P*-value is also given on the plots.

The results from the two-sample Mendelian randomization in the related Europeans were consistent with the one-sample results: there was evidence of a causal association in all individuals and in women only, but no consistent evidence in men (Figure 4 and Table 2;Supplementary Table 6, available as Supplementary data at *IJE* online). The estimates from the Egger- and Median-MR analyses were consistent, although confidence intervals were wider. The Egger method suggested that no pleiotropy was present (Egger intercept *P*-value: 0.41 in all individuals). We obtained very similar estimates when performing sensitivity analyses, including using different definitions of depression and excluding underweight (<18.5 kg/m^2^) individuals, those with other health conditions, disease or parental depression, and including all 76 BMI variants in the GRS (Supplementary Table 7, available as Supplementary data at *IJE* online).

###### Meta-analysis with results from the Psychiatric Genomics Consortium

**Figure 5.**
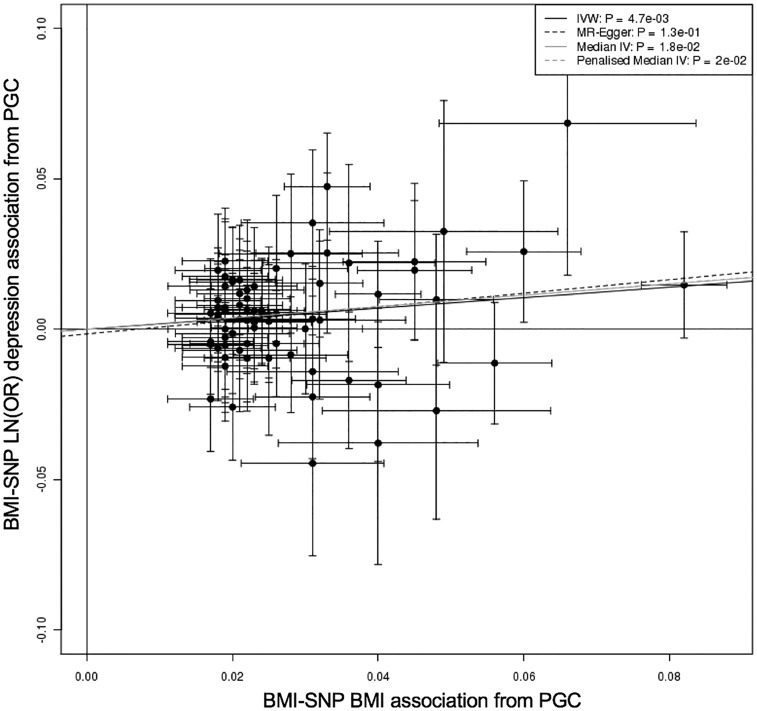
Plot of the individual BMI variant—BMI associations from the primary GWAS that did not include UK Biobank^22^ against the BMI variant—depression associations on natural log scale (LN(OR)) from the PGC GWAS data excluding the UK Biobank. The beta regression coefficients for inverse variance weighted (IVW) instrumental analysis (black solid), Egger-MR (black dash),^33^ median-IV (grey solid) and the penalized weight median IV (grey dash) are plotted.

To provide further evidence for or against the causal role of BMI in depression in all individuals, we next used the summary statistics from the PGC data, which included 45 591 depression cases and 97 647 controls, excluding the UK Biobank.^15^ We performed two-sample MR which provided evidence of a causal role for BMI in major depressive disorder. A 1-SD higher BMI was associated with 1.19 (95% CI: 1.06, 1.34) higher odds of MDD (*P *=* *0.005; Figure 5; and Supplementary Table 5, available as Supplementary data at *IJE* online). Similar associations were noted using methods which are more robust to pleiotropy, although the estimate from MR-Egger crossed the null (Supplementary Table 6, available as Supplementary data at *IJE* online). Meta-analysis of the MR-Egger estimate from the UK Biobank and the PGC suggested a causal relationship with a 1-SD higher BMI resulting in 1.25 (95% CI: 1.03, 1.51) higher odds of depression, with no evidence of heterogeneity (*P *=* *0.96).

###### Neuronal and non-neuronal BMI genetic instruments

We next tested whether the causal association between BMI and depression was consistent when using genetic instruments for BMI that reflected neuronal versus non-neuronal pathways. Evidence for a role of neuronal pathways in depression was stronger than for non-neuronal pathways. A 1-SD genetically higher BMI, as defined by neuronal and non-neuronal GRS, was associated with a 1.26 (95% CI: 1.07, 1.49, *P *=* *0.013) and 1.08(95% CI: 0.93, 1.26, *P = *0.31) higher odds of depression respectively (*P*_difference_ = 0.17). Similar estimates were obtained with the PGC data (neuronal: 1.24, 95% CI: 1.06, 1.45, *P *=* *0.010; non-neuronal: 1.14, 95% CI: 0.94, 1.38, *P *=* *0.21), and meta-analyses of the UK Biobank and PGC estimates suggested no difference between the neuronal and non-neuronal instruments (*P*_heterogeneity_ = 0.14).

### Favourable adiposity

Meta-analysis of the inverse-variance weighted Mendelian randomization estimates from UK Biobank and PGC data provided tentative evidence of a causal relationship between favourable adiposity and depression (OR: 1.26, 95% CI: 1.06, 1.50, *P *=* *0.010). The estimates did not reach *P* <0.05 in the UK Biobank or PGC studies^15^ separately, but there was no evidence of heterogeneity. The confidence limits included the causal estimate from the standard ‘unfavourable adiposity’ BMI instrument (Table 2; and Supplementary Table 8, available as Supplementary data at *IJE* online).

#### Childhood BMI and depression later in life

We next tested the association of childhood adiposity with adult depression. We used self-reported perceived body size at age 10, and validated these self-report measures by testing their association with the 73 SNP BMI GRS. Genetic variants associated with BMI tend to have similar effects in childhood, suggesting that they act as an instrument for exposure to higher BMI throughout most of life including childhood.^40^ In line with this assumption, the BMI GRS was associated with self-reported size at age 10 in the directions expected (Supplementary Table 9, available as Supplementary data at *IJE* online). When comparing with individuals who reported being of average body size at age 10, individuals who perceived themselves as plump at age 10 had higher odds of reporting depression (OR: 1.32, 95% CI: 1.28, 1.37; Supplementary Table 10, available as Supplementary data at *IJE* online). Similarly, individuals who perceived themselves as thinner than average at age 10 also had higher odds of reporting depression than individuals who were of average size at age 10 (OR: 1.20, 95% CI: 1.17, 1.23). These associations were similar in men and women and when adult BMI was included as a covariate (Supplementary Table 10, available as Supplementary data at *IJE* online).

### Negative control Mendelian randomization tests

We next selected three variables as negative control tests—variables that would be very unlikely to be a consequence of higher BMI and would help control for any residual confounding in the data which could affect genetic estimates in such large sample sizes. These were higher nitrogen dioxide pollution levels at home location, urban versus rural dwelling and less frequent sun protection use in the UK Biobank, all of which were strongly observationally associated with higher BMI, most likely as a result of the association between higher BMI and poverty and lower educational attainment (Table 3). We observed some nominal evidence of a causal association between higher BMI and these variables, but the inclusion of a measure of socioeconomic position, previously shown to be potentially a consequence of lower BMI, especially in women,^38^ attenuated the associations (Table 3). Adjusting for socioeconomic position did not alter the causal relationship between BMI and depression.

**Table 3.** Associations between higher BMI and three negative control variables, using regression and instrumental variable analysis and based on the unrelated individuals used in the one sample MR. Our depression variable is included for reference

		Observational^b^		Genetic^c^		Genetic^d^	
Negative control variable	*N*	Beta or LN(OR)^e^ representing change in negative control variable or depression per SD higher BMI	*P*	Beta or LN(OR)^e^ representing change in negative control variable per SD higher genetically instrumented BMI	*P*	Beta or LN(OR)^e^ representing change in negative control variable per SD higher genetically instrumented BMI	*P*
Regular sun protection use^a^	375 720	−0.041 (−0.047, −0.036)	<1 x 10^−15^	−0.052 (−0.098, −0.006)	0.028	−0.040 (−0.087, 0.006)	0.09
Nitrogen dioxide pollution level	372 791	0.023 (0.020, 0.026)	<1 x 10^−15^	0.025 (0.000, 0.050)	0.050	−0.006 (−0.030, 0.018)	0.63
Rural dwelling	386 131	−0.067 (−0.076, −0.058)	<1 x 10^−15^	−0.023 (−0.094, 0.048)	0.52	0.006 (−0.065, 0.077)	0.88
Depression	41 397 (246 106)	0.150 (0.139, 0.160)	<1 x 10^−15^	0.166 (0.084, 0.247)	7 x 10^−5^	0.153 (0.07, 0.235)	0.0002

a Coded as never, sometimes, most of the time, always. Analysed using ordinal logistic regression.

b Regression analysis adjusting for age and sex.

c One-sample instrumental variable analysis with a BMI GRS.

d One-sample instrumental variable analysis with a BMI GRS and accounting for socioeconomic position using the Townsend deprivation index.

e On natural log scale, LN(OR).

## Discussion

Using genetic variants as unconfounded proxies for BMI, our study provides further evidence that higher BMI, and therefore obesity, leads to higher odds of depression.^12–15^^,^^41^ Several of our sensitivity analyses did not reach formal levels of statistical significance, but replication by the independent studies of the PGC provided additional evidence of a causal effect from higher BMI to higher depression. In addition, we investigated the causal relationship between higher BMI and depression in the absence of adverse metabolic effects, investigated the relationship in men and women individually, and demonstrated the causal relationship between a binary measure of obesity and depression.

This study adds to evidence that higher BMI or obesity causes depression, which has been suggested in previous observational and genetic studies. However, we have not ruled a possible bidirectional causal relationship between higher BMI and depression, as suggested by earlier systematic reviews of longitudinal studies.^11^ Further research is required to explore the causal role of depression on body mass index and obesity. The power for testing the relationship from depression to BMI is currently limited, but as more genetic variants are identified for depression this relationship can be more comprehensively explored.

The availability of individual-level data in the UK Biobank allowed us to test a number of hypotheses not easily performed in the context of a GWAS consortium, most notably the stratification by sex. Our study highlighted potential sex differences in the causal relationships between BMI and depression which are consistent with observational data, with stronger associations in women, although even larger sample sizes or stronger genetic instruments are needed to confirm this difference. The prevalence of depression is consistently higher in women than men,^42^ and in the UK Biobank women were 1.5 times more likely to report a diagnosis of depression. We have previously shown, using a Mendelian randomization approach, that high BMI is causally associated with lower socioeconomic position in women but with no such evidence in men.^38^ The combination of these MR results suggests that that the stigma of high BMI differs between the sexes, and may reflect a causal pathway from higher BMI to lower socioeconomic position to higher depression.

A range of factors could link higher BMI to higher odds of depression, including poorer general health as a result of high BMI or psychological impacts of obesity. To investigate this in more detail, we used a genetic instrument for ‘favourable adiposity’, where alleles associated with higher BMI are associated with lower risk of type 2 diabetes, and lower insulin levels. Our analyses provided some tentative evidence for a causal relationship between favourable adiposity and depression. This analysis was important in that it suggests a psychosocial effect of higher BMI as well as, or instead of, a physiological effect driven by adverse metabolic health.

We also split the BMI GRS into two risk scores, one with genetic variants acting through neuronal pathways and the other with genetic variants with non-neuronal functions and, whereas the association was stronger with neuronal BMI variants, the confidence intervals for the two genetic risk scores overlapped. The overlap in the confidence intervals meant that we were not able to state confidently that there was a difference in the association with depression between the neuronal and non-neuronal BMI variants.

Most of the genetic variants we used as an instrument for BMI reflect lifelong differences in BMI, and the association of the BMI GRS with self-perceived measure of body size at age 10 years suggested it is a valid measure of BMI in childhood. Although this suggests that the BMI instrument is in part testing the exposure to higher BMI from early childhood, we cannot use this current list of genetic variants to dissect the causal role of lon- term exposure to higher BMI from more rapid weight gain later in adulthood. Nevertheless, in observational analyses, individuals who perceived themselves as plumper than average at age 10 had higher odds of depression in adulthood than those who reported average body size at age 10. This association is consistent with evidence from longitudinal studies suggesting that childhood obesity increases the risk of adulthood depression.^43^^,^^44^ In our analyses, individuals who reported to be thinner than average at age 10 were also more likely to report depression than those individuals reporting normal body size at age 10, which may help explain the U-shaped relationship between BMI and depression,^5^ but could also be confounded by a range of factors including poverty.

### Strengths and limitations

The major strength of this study was the availability of individual-level data in more than 40 000 depression cases and 300 000 controls. This allowed us to perform sex-specific analyses and a number of crucial sensitivity analyses, to provide a comprehensive investigation for the causal role of BMI, and different aspects of BMI, in MDD. We acknowledge a number of limitations with this study. First, the UK Biobank participants were born between 1938 and 1971, and the causal associations may not apply to younger birth cohorts or be generalizable outside the UK. However, our results were consistent with several other studies which used data from individuals of different ages and from different European countries^14^^,^^15^ and PGC data excluding the UK Biobank. Second, the definition of depression was not gold standard; we used both Hospital Episode Statistic data and derived self-reported data to identify depression cases. The experience of some level of depressive symptoms is part of human experience, and therefore it is challenging to define clinical depression cases and controls based on self-reported data. Additionally, the episodic nature of depression makes it difficult to define incident depression; therefore in this study we looked at any depression rather than prevalent versus incident. However, our results were consistent with previous studies and when only MDD defined from the Hospital Episode Statistics data in the UK Biobank was used. Third, the BMI GRS was associated with smoking. However, this association was minimal in comparison with the strength of the association with raw BMI, and unlikely to be altering the results of this study. Fourth, the BMI GRS we used is likely to be an instrument to exposure to high BMI from childhood to adulthood because it is associated with BMI at age 10 as well as at age 40 to 70 years. The use of a genetic risk score that captures BMI across a wide age range has advantages and limitations. The advantage is that we capture high BMI for a long period of people’s lives, in contrast to the cross-sectional association of measured BMI from a single time point. The limitation is that we cannot separate potential causal effects of high BMI in adulthood from those in childhood. Fifth, the favourable adiposity GRS only explains a small percentage of the variance in BMI, but the large number of depression cases in the UK Biobank and PGC data meant we had sufficient power to detect a 1.23 higher OR per 1-SD higher BMI, an effect only slightly higher than that of the observational BMI-depression association. As more favourable adiposity variants are identified, we will be able to investigate this question in more detail. Finally, the one-sample approaches in the unrelated individuals may not fully account for genetic population differences.^45^ However, our negative control results, using sun protection, air pollution and home location, demonstrated no causal relationships with the BMI genetic risk score when socioeconomic status was accounted for, and our analyses in the related sample using the linear mixed model provided consistent estimates.

In summary, using up to 340 000 participants from the UK Biobank, we provide evidence that higher BMI, as estimated by genetics is causally related to higher odds of depression, especially in women.

## Funding

J.T. is funded by a Diabetes Research and Wellness Foundation Fellowship. A. Mulugeta studentship is funded by Australian Research Training Program Scholarship. S.E.J. is funded by the Medical Research Council (grant: MR/M005070/1) M.A.T., M.N.W. and A. Murray are supported by the Wellcome Trust Institutional Strategic Support Award (WT097835MF). A.R.W. and T.M.F. are supported by the European Research Council grant: SZ-245 50371-GLUCOSEGENES-FP7-IDEAS-ERC. R.M.F. is a Sir Henry Dale Fellow (Wellcome Trust and Royal Society grant: 104150/Z/14/Z). R.B. is funded by the Wellcome Trust and Royal Society grant: 104150/Z/14/Z. K.S.R. is supported by funding from the Gillings Family Foundation. H.Y. is funded by Diabetes UK RD Lawrence fellowship (grant: 17/0005594). C.M.L. was funded in part by the National Institute for Health Research (NIHR) Biomedical Research Centre at South London and Maudsley NHS Foundation Trust and King’s College London. The views expressed are those of the author(s) and not necessarily those of the NHS, the NIHR or the Department of Health and Social Care.

## Supplementary Material

dyy223_Supplementary_Data
